# Dentition and Its Derangements

**Published:** 1862-08

**Authors:** A. Jacobi


### Selections.

DENTITION AND ITS DERANGEMENTS.—*By A. Jacobi*, M. D. *LECTURE IX*—PART 3.—You will find that the majority of such remarks as I shall have to make on the connection between dentition and eczema are also due to impetigo. I therefore shall speak of eczema alone, expecting to add a few words, towards the end of this lecture, on the latter eruption.

Eczema is a very frequent cutaneous disease, and again, like so many others, consists in a superficial dermatitis, serous exudation taking place on the surface of the cutis. It takes no typical or regular course like most forms of herpes, and spreads over a larger surface than herpes generally does. In this respect, it has been compared to catarrh on the mucous membrane, as in this form of disease also the surface is more affected than the subjacent tissue, the secretions being also copious and spreading considerably and rapidly. In the majority of cases the epidermis is lifted up by the exudation of eczema, and vesicles are formed; not always, however, in the like manner. In the simple eczema, *e. simplex*, or *vesiculosum*, the vesicles are of small size, and not surrounded by inflammation, and extend generally over a large surface. The contents of the vesicles are entirely clear and transparent. In the *e. impetiginosum* the contents are less transparent, more similar in purulent matter, a formation of young cells having commenced in the hitherto liquid and uniform exudation. In another form, *pityriasis rubra,* the exudation is not copious, not even enough so to form vesicles, but it will dry up soon and form scales on the hyperæmic surface. In *e. rubrum* (salt rheum) a similar process takes place; no epidermis is lifted up, no vesicle is formed, and the cutis is denuded of its epidermis, hyperæmic and moist. In *tinea* (of the head, face, ears, lips, extremities, etc.), or *crusta lactea, milk crust,* the exudation is copious enough to form vesicles, the contents of which become dry and hard, and with the admixture of the epidermical scales and extraneous accidental matter, form hard, and more or less thick scales on the surface. Sometimes, also, a thickening of the adjacent cutis takes place, like the swelling of the submucous tissue in chronic catarrhal affections of a mucous membrane.

Crusta lactea, then, is a chronic eczema, and generally not universal, but mostly confined to a single region of the surface, scalp, face, lips, etc. It is almost always a troublesome and tedious affection, is very frequently observed in children, in their first year and later, and frequently believed to be in a direct connection with dentition. On the scalp (*e. capillitii*) the two forms, *impetiginosum* and *rubrum,* are most common. Vesicles are soon formed, but as fingers and combs will be equally busy in destroying them, they are frequently not noticed. The scalp, then, feels moist to the touch, the hair appears to be glued together, the secretion dries up, and scabs are formed, which together with new secretion, and the bundles of wet and sticky hair, prove a safe depot for dirt of any sort, and a pet refuge for lice. At the same time the cervical glands are found swelled, and even suppurating. This form of eczema, in which hard or soft, thick or thin scales cover the scalp in the described form, was formerly known by the name of *granulated tinea favosa,* while that form, which, as *pityriasis rubra,* consisted in the formation and ejection of a large number of small epithelial scales, was called *tinea furfuracea.*

In children, eczema is almost as frequent on the face as on the scalp. There, again, it assumes the same forms of *e. impetiginosum* and *e. rubrum.* Cheeks and chin appear red, have a brilliant appearance, and after a few small vesicles have been formed and broken, are moist and assume a yellowish color, beneath which the corium is found denuded and moist with serum. It is not necessary to allude to all the names formerly given to different forms and localities of this affection. It is enough to say that the same eruption will show itself on the external ear, on the eyelids, nose and lips, on the shoulders, and almost all the surface of the body, very generally complicated with swellings of the neighboring glands, and always proving, as I have stated already, either a very obstinate eruption, as far as its removal is concerned, or at all events one that is very apt to return after it has been once removed.

We shall approach our subject a little nearer by examining the causes of the eczematous eruptions, for the purpose of ascertaining how far we are justified in assuming a causal relation between eczema and dentition. Immediate irritation of the skin stands foremost among the causes of eczematous eruptions; hot temperature from whatever source, *e. caloricum*, influence of the sun, *e. solare*, and of warm baths, and frequently also after repeated cold baths, alternating with warm ones, etc. The "critical eruptions" of hydropathists, so much boasted of by busy doctors, and so much believed in by credulous patients of the "water-cure establishments," belong to this class. The embrocation of salves, especially mercurial, irritation by scratching, and the presence of parasitic animals on or within the skin, are also frequent causes of eczema. Many of the vesicles and pustules found on the skin during the presence of itch are more due to the busy working of the patient's fingers than to the direct influence of the acarus scabiei. Another cause of eczema authors are in the habit of describing for such forms which are found especially on the lower extremities; they are the result of obstruction in the circulation of the venous blood, and but rarely found except in advanced age, i. e. those years of life in which venous obstructions are common and best known in the form of "abdominal plethora," chronic catarrhs of the stomach and intestines, and hæmorrhoidal swellings of the veins of the rectum. We have to exclude this form from our list, as it is not found in infantile age. But there are other causes of eczema which concern us more than almost any others. The intimate connection of the skin with all the other organs of the system includes the necessity of its being directly influenced by internal sufferings of any kind; chronic eruptions are, therefore, the frequent results of constitutional affections, and eczema from such causes is a frequent occurrence. Especially such children as show an excessive liability to exorbitant deposits of fat in the subcutaneous tissue, and those affected with symptoms of rhachitis and scrofula, appear to be the proper individuals for obstinate chronic eczema. This connection between eczema, scrofula, and rhachitis is as frequent and sure as that between inflammations of the submucous tissue and scrofula, and between rhachitis and bronchial or intestinal catarrhs. Eczema, therefore, is an almost regular companion of such children as have a white skin and soft and silk-like, fair hair, whose bones are backward in their development, whose occiput is soft, and dentition little advanced, who suffer from bronchial and intestinal catarrh (the latter in the forms either of diarrhea or costiveness), who are cross, peevish, and fretful, show slight symptoms of convulsions while remaining in their general state of health, and sudden exclamations in their sleep, and who, at the same time, show other symptoms of disorder in the organs of digestion, assimilation, and elimination, viz., large stomach, occasional colics and vomiting, and the presence of large amounts of phosphate of lime in the urine. After all, there is no doubt that large numbers of cases of eczema depend on constitutional causes; as is well known, also, that in adult females, eczema is a frequent attendant of chlorosis and other anomalies of menstrual secretion. Even hereditary disposition can not be entirely denied as one of the causes of eczema, but we have no reason whatever, after the investigations of Hebra, of Vienna, to further believe in the presence of a "herpetic diathesis" in all such cases of eczema which appear unexplainable by either a local or general cause.

I have stated that some cutaneous diseases must be considered as merely local affections, while others are in direct connection with internal diseases of either local or general character. That, however, dentition, as such, should be the cause of obstinate eczema, I have had no reason to say. It is true that, during the period of dentition, viz., from the sixth to the thirtieth month, eczema is a very common occurrence. Not only, however, will eczema be found before this period, and very frequently so after the third or fourth month, when improper food has had time to show its effects, and irregular development of the cranium, brain, and scalp becomes visible in a number of symptoms; but within the mentioned time fall rhachitis, and the first symptoms of scrofula. The physiological facts to which I have frequently had to resort for the purpose of explaining many symptoms attributed to dentition, i e. the rapid development of the cranial bones and cavity, with the integuments, appear fully to explain why it is, that such children who suffer from hereditary or other predisposition will suffer from cutaneous affections on these parts which just are the subjects of the most rapid, though in itself normal, development.

The presumed connection of eczema of the head and face with dentition has been one of the causes why the treatment of this affection has been neglected as being useless, and its cure condemned as injurious. Not to speak of the supposed or real relation between the same affection and the diseases of internal organs, which also has induced most authors and practitioners to consider eczema of the head and face, at certain times of infantile development, as a *noli me tangere.* This is justified, as far as diseases are concerned, to a certain extent. Good observers have noticed that soon after a sudden suppression of eruptions on the scalp, acute hydrocephalus, laryngitis, bronchial catarrh, and intestinal catarrh would show themselves, while also severe cases of bronchial catarrh would get well after the extensive appearance of eczema of the scalp and face. Nobody can prove, to a certainty, the dependency of these facts on each other, but they show enough to give the impression of there being at least the possibility of a causal correlation between them. At the same time it is desirable to remember, that the sudden suppression of a physiological or pathological secretion, to which the system had been accustomed for a long period, has been observed to be followed by a morbid secretion on some distant part of the organism. As a single instance, I allude to the serous secretion of the mucous membrane of the intestinal tract after extensive combustion of the skin ; and it may well be possible that the suppression of an extensive and long continued secretion on the scalp may give rise to internal transudations. Thus, while I consider the majority of the cases of eczema, as far as they do not depend on rhachitis and scrofula, as of a mostly local character, I am myself averse, not to removing cranial and facial eczema, but to removing it suddenly. By slowly suppressing a protracted secretion of this kind, we by no means disturb the equilibrium of circulation, to any great extent.

To be complete, however, on this very important subject, I ought to add, that our principal modern authority on diseases of the skin, Prof. Hebra, of Vienna, is entirely opposed to considering cutaneous affections of this sort as anything but local. He first urges this point, that the local remedies applied to the skin are by no means local applications only. For it is true that the skin is not only the receiving organ for a number of other diseases (syphilis, hydrophobia, and animal poisons generally), but also, that the uninjured epidermis allows a number of medicaments, mercury, iodine, quinine, to enter the system. At all events, then, cutaneous affections are not repelled into the system by local applications, to start again on other forms and on other places, but the effect may justly be considered a general one. Be this as it may, Prof. Hebra discards entirely all and every one of the hæmato-cathartic—blood purifying—medicines, purgatives, drastics, herbs, antimonials, baryta, graphyta, sulphur, cantharides, mezereum, viola tricolor, dulcamara, Roob de Laffecteur, Larrey's Syrup, etc. The only internal medicines he ever uses are arsenic, mercurials, iodine, iron, cod-liver oil, amara, acids and all such remedies as would have been given in the same case for the dryscrasic affection, no matter whether it was accompanied by a local affection or not.

I have deemed it proper to deviate a little from my subject to give in full the general views on the advisability and practicability of treatment and cure, in case of local cutaneous affections. As to local remedies used by myself in cases of eczema of the head and face, I will briefly add that they are principally alkalies, such as potassa, astringents such as zinc, tannin, and lead, and "alterants," such as white or red precipitate of mercury, saving for my future lectures on the diseases of the skin, as a subject for further investigation, the exposition of their individual indications.

On *impetigo* I have but a few words to say. Originally it is identical with eczema; but it has darker and thicker scabs, and the vesicles show white or yellowish contents. Below the scabs there is generally to be found a collection of purulent matter. I have already stated that there is a transitory development of the eczematous vessels into the impetigonous pustule in *eczema impeciginosum*, consisting in the formation of a number of young cells in the transparent contents of the eczematous vesicles. That form in which this process commences early, and in general is called impetigo. Therefore, it is not improper, in my opinion, which, however, I do not claim as original, to consider eczema and impetigo as one and the same affection, the number of cells that have been formed depending on the vulnerability and general condition of the skin, and general scrofulous disposition. Impetigo, together with glandular swellings and chronic catarrh, are the principal symptoms by which scrofula is identified. As this is so, this form of cutaneous affection deserves greatly more than the former, a general treatment directed against the original disposition and morbid constitution. That dentition is entirely out of the question, as a direct cause of impetigo, I need not add.—*Am. Med. Times.*

